# Vitamin D and Delirium in Older Adults: A Case-Control Study in Geriatric Acute Care Unit

**DOI:** 10.3389/fneur.2020.01034

**Published:** 2020-09-18

**Authors:** Justine Chouët, Guillaume Sacco, Spyridon N. Karras, David J. Llewellyn, Dolores Sánchez-Rodríguez, Cédric Annweiler

**Affiliations:** ^1^Department of Geriatric Medicine, Cholet Hospital, Cholet, France; ^2^Department of Geriatric Medicine, Angers University Memory Clinic, Research Center on Autonomy and Longevity, Angers University Hospital, Angers, France; ^3^UPRES EA 4638 and Health Faculty, University of Angers, Angers, France; ^4^Division of Endocrinology and Metabolism, First Department of Internal Medicine, Medical School, Aristotle University of Thessaloniki, AHEPA University Hospital, Thessaloniki, Greece; ^5^University of Exeter Medical School, Exeter, United Kingdom; ^6^WHO Collaborating Centre for Public Health Aspects of Musculoskeletal Health and Aging, Division of Public Health, Epidemiology and Health Economics, University of Liege, Liege, Belgium; ^7^Department of Medical Biophysics, Robarts Research Institute, Schulich School of Medicine and Dentistry, the University of Western Ontario, London, ON, Canada

**Keywords:** vitamin D, delirium, cognition, older adults, neuroendocrinology

## Abstract

**Objective:** Vitamin D is involved in brain health and function. Our objective was to determine whether the serum 25-hydroxyvitamin D (25OHD) concentration was associated with delirium in a case-control study of geriatric inpatients.

**Methods:** Sixty cases with delirium (mean ± SD, 84.8 ± 5.7years; 58.3% female) and 180 age- and gender-matched controls were enrolled in a geriatric acute care unit between 2012 and 2014. The diagnosis of delirium was made using the Confusion Assessment Method. Hypovitaminosis D was defined using consecutively the consensual threshold value of 50 nmol/L and a threshold value calculated from a sensitivity-specificity analysis. Age, gender, number of acute diseases, use of psychoactive drugs, season of testing, and serum concentrations of calcium, parathyroid hormone, creatinine, albumin, TSH, vitamin B9 and vitamin B12 were used as potential confounders.

**Results:** The 60 cases with delirium exhibited lower 25OHD concentration than 180 matched controls (35.4 ± 30.0 nmol/L vs. 45.9 ± 34.5 nmol/L, *p* = 0.035). Increased 25OHD concentration was associated with a decrease in delirium prevalence (OR = 0.99 [95CI: 0.98–0.99] per nmol/L of 25OHD, *p* = 0.038). The concentration distinguishing between cases and controls with the best sensitivity-specificity was found between 29.5 and 30.5 nmol/L. The regression models showed that delirium was associated with hypovitaminosis D defined either as 25OHD ≤ 50 nmol/L (OR = 2.37 [95CI: 1.07–5.25], *p* = 0.034) or as 25OHD ≤ 30 nmol/L (OR = 2.66 [95 CI: 1.30–5.45], *p* = 0.008).

**Conclusions:** Decreased serum 25OHD concentrations were associated with delirium among acute geriatric inpatients. The threshold concentration to differentiate between cases and controls was around 30 nmol/L.

## Introduction

Clinical importance of hypovitaminosis D is linked to its high prevalence, estimated at more than one billion people around the word (1), and its health manifestations. Vitamin D is classically known for its regulatory function in phosphocalcic metabolism (2). A growing number of studies have also reported non-bone effects of vitamin D in the past decade, especially on brain health and function (3–5).

Hypovitaminosis D has been repeatedly associated with neurocognition (6), particularly with major neurocognitive disorders (7, 8) and accelerated cognitive decline (8, 9). However, epidemiological associations reported thus far mainly implied chronic neurocognitive disorders. In contrast, acute neurocognitive disorders such as delirium were only scarcely examined in this context.

Delirium is an acute neurocognitive disorder leading to greater morbidity (pressure ulcers, infectious diseases, fall risk, disability), complicated care course (prolonged hospital stays, more frequent readmissions, increased healthcare costs), and greater mid-term mortality (10–12). Pathophysiology of delirium remains not fully elucidated. Some risk factors are well identified, including the advance in age, male gender, history of chronic neurocognitive disorders or psychiatric conditions (11). However, these risk factors are unchangeable and cannot be targeted to prevent delirium. Thus, potential changeable risk factors are the matter of extensive research. Several hypotheses are proposed, mainly based on biological imbalance within the central nervous system (11). Considering the neurocognitive effects of vitamin D, we hypothesized that hypovitaminosis D could participate to the onset of delirium among older adults. The aim of the present study was to determine whether serum 25-hydroxyvitamin D (25OHD) concentration was associated with delirium among geriatric inpatients hospitalized in an acute care unit.

## Methods

### Design and Settings

We conducted a unicentric case-control study (1 case with delirium for 3 controls without delirium) in the geriatric acute care unit of the University Hospital of Angers, France, between 2012 and 2014. At the admission into the unit, participants received standardized clinical and neuropsychological assessments together with a blood test. The study was approved by the local ethics committee and the Commission nationale informatique et libertés (number 1408714).

### Participants

All patients aged 70 years and older consecutively admitted between 2012 and 2014 in the unit were considered for inclusion in the study. Inclusion criterion for cases was the diagnosis of delirium according to the Confusion Assessment Method (CAM) (13). Controls exhibiting no diagnosis of delirium according to the CAM were matched by age and gender (ratio 3:1). All participants were affiliated to the French social insurance.

### Data Collection

#### Delirium

Delirium was identified using the CAM (13), a standardized tool aiming to screening for delirium. A senior physician assessed four main criteria (a) acute onset and fluctuating course, (b) inattention, (c) disorganized thinking, (d) altered level of consciousness. Delirium was retained when criteria (a) and (b) associated to criteria (c) and/or (d) were retrieved. This assessment is quick and easy to use with good metrological performance (13, 14).

#### Serum 25-hydroxyvitamin D Concentration

Serum concentrations of 25OHD effectively reflect the stock of vitamin D in the body. Serum concentrations of 25OHD were measured at the time of the CAM test by radioimmunoassay (DiaSorin Inc., Stillwater, MN) in nmol/L (to convert to ng/mL, divide by 2.496). With this method, there is no interference with lipids, which is often observed in other non-chromatographic assays of 25OHD. The intra- and interassay precision were 5.2 and 11.3%, respectively. Hypovitaminosis D was successively defined using either the consensual threshold value of 50 nmol/L according to the World Health Organization and the National Institutes of Health definitions, or using a threshold value calculated from a sensitivity-specificity analysis to best fit the studied group.

#### Covariates

Age, gender, number of acute diseases, use of psychoactive drugs, season of testing, serum concentrations of calcium, parathyroid hormone, creatinine, albumin, thyroid-stimulating hormone (TSH), vitamins B9 and B12 were used as potential confounders. The number of acute diseases was assessed by a senior physician at the admission. Using psychoactive drugs (i.e., antidepressants or neuroleptics or anxiolytics) were determined from drug prescriptions at the admission. The season of testing were categorized as follows: Spring from March 21 to June 20, Summer from June 21 to September 20, Autumn from September 21 to December 20, and Winter from December 21 to March 20. All blood samples were analyzed using standardized laboratory methods at the University Hospital of Angers, France.

### Number of Participants

Because the main objective of the study was descriptive, no calculation of number of subjects was required. However, to match a normal distribution of the data and to use parametric statistical tests, at least 30 participants were required in each group (i.e., *n* = 30 participants with delirium and *n* = 30 controls at least). To improve the power of the study, we chose to triple the number of controls (i.e., 3 controls per 1 case).

### Statistics

The participants' characteristics were summarized using means and standard deviations (SD) or frequencies and percentages, as appropriate. Normality of data distribution was checked using skewness-kurtosis test. As the number of observations was higher than 40, comparisons were not affected by the shape of the error distribution and no transform was applied (15). Firstly, comparisons between cases with delirium and controls without delirium were performed using Student's *t*-test or the Chi-square test, as appropriate. Secondly, the serum 25OHD threshold value that best distinguished between cases and controls was determined by sensitivity analysis. Thirdly, a multiple logistic regression was used to examine the association between delirium (dependent variable) and serum 25OHD (independent variable), while adjusting for potential confounders. Finally, similar logistic models were used to examine the association between delirium and hypovitaminosis D defined either using the consensual threshold value of 50 nmol/L or using the threshold value calculated with the sensitivity analysis. *P*-values < 0.05 were considered significant. All statistics were performed using SPSS (v19.0, IBM Corporation, Chicago, IL).

## Results

Sixty cases with delirium (mean ± SD, 84.8 ± 5.7years; 58.3% female) and 180 controls without delirium (84.8 ± 5.7 years; 58.3% female) were included in the study. The mean serum 25OHD concentration was 43.3 ± 33.7 nmol/L, and 67.5% of the studied group exhibited hypovitaminosis D ≤ 50 nmol/L. Serum 25OHD concentration was lower among cases compared to controls (respectively, 35.4 ± 30.0 nmol/L vs. 45.9 ± 34.5 nmol/L, *p* = *0.035*), and hypovitaminosis D ≤ 50 nmol/L was also more frequent among cases compared to controls (*p* = 0.039) (Table 1).

**Table 1:** Participants' characteristics and comparisons between cases with delirium (*n* = 60) and age- and gender-matched controls without delirium (*n* = 180).

		**Delirium**		
	**Total (*n* = 240)**	**Yes (*n* = 60)**	**No (*n* = 180)**	*P*-value^*^
**Clinical and demographical measures**				
Age, years	84.8 ± 5.7	84.8 ± 5.7	84.8 ± 5.7	1.000
Female gender, *n* (%)	140 (58.3)	35 (58.3)	105 (58.3)	1.000
Number of acute diseases at the admission	8.5 ± 4.6	9.8 ± 4.5	8.1 ± 4.6	**0.012**
Use of psychoactive drugs, *n* (%)	95 (39.7)	27 (45.0)	68 (38.0)	0.337
Season of testing				
Spring, *n* (%)	58 (24.2)	14 (23.3)	44 (24.4)	0.648
Summer, *n* (%)	50 (20.8)	10 (16.7)	40 (22.2)	
Autumn, *n* (%)	73 (30.4)	18 (30.0)	55 (30.6)	
Winter, *n* (%)	59 (24.6)	18 (30.0)	41 (22.8)	
**Serum measures**				
25OHD, nmol/L	43.3 ± 33.7	35.4 ± 30.0	45.9 ± 34.5	**0.035**
Hypovitaminosis D^*^, *n* (%)	162 (67.5)	47 (78.3)	115 (63.9)	**0.039**
Calcium, mmol/L	2.26 ± 1.15	2.26 ± 0.14	2.26 ± 0.15	0.866
Parathyroid hormone, pg/mL	43.2 ± 35.4	46.0 ± 22.6	42.3 ± 38.7	0.498
Creatinine, μmol/L	94.0 ± 59.1	94.8 ± 63.1	93.8 ± 57.9	0.907
Albumin, g/L	34.1 ± 5.5	35.5 ± 5.5	33.6 ± 5.5	**0.023**
TSH, mUI/L	1.70 ± 1.79	1.84 ± 1.69	1.66 ± 1.82	0.511
Vitamin B9, μg/L	6.7 ± 6.1	6.6 ± 4.3	6.7 ± 6.6	0.904
Vitamin B12, ng/L	447.3 ± 311.7	453.1 ± 234.3	445.4 ± 333.9	0.873

Quantitative data are presented as mean ± standard deviation; 25OHD, 25-hydroxyvitamin D; TSH, thyroid-stimulating hormone;

* *serum 25-hydroxyvitamin D ≤ 50 nmol/L; p-value significant < 0.05 indicated in bold*.

Multiple logistic regression models showed that the increase in serum 25OHD was inversely associated to delirium (OR = 0.989 [95% confidence interval (CI): 0.979–0.999] by nmol/L of 25OHD, *p* = 0.038), even after adjusting for potential confounders (OR = 0.988 [95CI: 0.975–0.999] by nmol/L of 25OHD, *p* = 0.047) (Table 2).

**Table 2:** Univariate and multiple logistic regression models assessing the cross-sectional association between serum 25-hydroxyvitamin D concentration (independent variable) and the diagnosis of delirium (dependent variable) after adjustment for potential confounders^*^ (*n* = 240).

	**Delirium**			
	**Unadjusted model**		**Fully adjusted model**	
	**OR [95% CI]**	*p*-value^*^	**OR [95% CI]**	*p*-value^*^
Serum 25OHD concentration	0.989 [0.979–0.999]	**0.038**	0.988 [0.975–0.999]	**0.047**
Age	1.00 [0.95–1.05]	1.000	1.00 [0.94–1.06]	0.989
Female gender	1.00 [0.55–1.81]	1.000	1.36 [0.67–2.76]	0.392
Number of acute diseases at the admission	1.08 [1.02–1.15]	**0.014**	1.11 [1.03–1.20]	**0.006**
Use of psychoactive drugs	1.34 [0.74–2.41]	0.338	1.28 [0.65–2.50]	0.476
Serum calcium concentration	1.19 [0.16–8.91]	0.866	0.32 [0.02–4.37]	0.395
Serum parathyroid hormone concentration	1.00 [0.99–1.01]	0.497	1.00 [0.99–1.01]	0.680
Serum creatinine concentration	1.00 [0.99–1.01]	0.907	1.00 [0.99–1.01]	0.369
Serum albumin concentration	1.07 [1.01–1.13]	**0.024**	1.12 [1.04–1.20]	**0.003**
Serum TSH concentration	1.05 [0.90–1.23]	0.513	1.07 [0.87–1.31]	0.532
Serum vitamin B9 concentration	1.00 [0.95–1.05]	0.903	1.00 [0.95–1.06]	0.877
Serum vitamin B12 concentration	1.00 [0.999–1.001]	0.872	1.00 [0.999–1.001]	0.467

25OHD, 25-hydroxyvitamin D; CI, confidence interval; OR, odds ratio; TSH, thyroid-stimulating hormone;

* *adjusted on season of testing without significant effect (OR = 1.00 [95CI: 0.73–1.35], p = 0.976); OR significant (i.e., p < 0.05) indicated in bold*.

The best compromise between sensitivity and specificity to predict delirium was calculated for a 25OHD threshold between 29.5 nmol/L (Se = 0.60, Sp = 0.37) and 30.5 nmol/L (Se = 0.58, Sp = 0.35).

Hypovitaminosis D was associated with delirium whether using the consensual threshold of 50 nmol/L (OR = 2.37 [95CI: 1.07–5.25], *p* = 0.034) or using the calculated threshold of 30 nmol/L (OR = 2.66 [95CI: 1.30–5.45], *p* = 0.008) (Table 3). Increased number of acute diseases and serum albumin concentration were also associated with delirium (Tables 2, 3).

**Table 3:** Multiple logistic regression models^*^ assessing the cross-sectional association between delirium and hypovitaminosis D defined using (A) consensual threshold of 50 nmol/L or (B) calculated threshold of 30 nmol/L (*n* = 240).

	**Delirium**			
	**Model A**		**Model B**	
	**OR** **[95% CI]**	***P*-value**	**OR** **[95% CI]**	***P*-value**
Consensual threshold 25OHD ≤ 50 nmol/L	2.37 [1.07–5.25]	**0.034**	–	–
Calculated threshold 25OHD ≤ 30 nmol/L	–	–	2.66 [1.30–5.45]	**0.008**
Age	1.00 [0.94–1.07]	0.935	1.00 [0.94–1.06]	0.967
Female gender	1.33 [0.66–2.70]	0.425	1.33 [0.65–2.71]	0.440
Number of acute diseases at the admission	1.11 [1.03–1.20]	**0.005**	1.11 [1.03–1.19]	**0.009**
Use of psychoactive drugs	1.35 [0.69–2.65]	0.376	1.34 [0.68–2.62]	0.399
Serum calcium concentration	0.38 [0.03–5.21]	0.467	0.30 [0.02–4.14]	0.372
Serum parathyroid hormone concentration	1.00 [0.99–1.01]	0.497	1.00 [0.98–1.01]	0.453
Serum creatinine concentration	1.00 [0.99–1.01]	0.776	1.00 [0.99–1.01]	0.249
Serum albumin concentration	1.12 [1.04–1.21]	**0.002**	1.12 [1.04–1.21]	**0.002**
Serum TSH concentration	1.07 [0.87–1.31]	0.524	1.07 [0.87–1.31]	0.538
Serum vitamin B9 concentration	1.00 [0.95–1.06]	0.909	1.01 [0.96–1.06]	0.776
Serum vitamin B12 concentration	1.00 [0.999–1.001]	0.493	1.00 [0.999–1.001]	0.596

25OHD, 25-hydroxyvitamin D; CI, confidence interval; OR, odds ratio; TSH, thyroid-stimulating hormone;

* *adjusted on season of testing without significant effect (OR = 1.02 [95CI: 0.75–1.39] with p = 0.886 in Model A; OR = 1.01 [95CI: 0.74–1.37] with p = 0.956 in Model B); OR significant (i.e., p < 0.05) indicated in bold*.

## Discussion

Delirium was associated with decreased 25OHD concentrations among older adults hospitalized in geriatric acute care unit, independently of all studied potential confounders. Hypovitaminosis D (defined either by the consensual threshold of 50 nmol/L or by a population-based threshold of 30 nmol/L) was associated with delirium.

Our results are in accordance with the scarce previous literature. Ford et al. found hypovitaminosis D (ie, insufficiency <75 nmol/L or deficiency <25 nmol/L) in 66% of people diagnosed with delirium from three Canadian university hospitals (12). Similarly, in a cohort of more than 4,000 patients, Quraishi et al. reported that serum 25OHD concentration <25 nmol/L prior to hospitalization was associated with an increased risk of delirium during the hospital stay (16), which suggested that the vitamin D status could be a changeable risk factor for delirium acquired during hospitalization. In contrast, such association was not found within intensive care units (17). The divergence could be explained by the initial severity of the acute medical problem and by the different levels of care provided, which may overpass the effect of hypovitaminosis D on delirium risk, if any. Compared to previous literature, we provide here the first case-control study on this issue to our knowledge, which allowed us to calculate the threshold of serum 25HOD corresponding specifically to the onset of delirium among geriatric inpatients.

Delirium is an acute cognitive dysfunction. Most previous studies reported that hypovitaminosis D could be associated with increased risks of Alzheimer disease (AD) and related disorders (18, 19). One meta-analysis found a 2.4-fold higher risk of cognitive dysfunction among people with hypovitaminosis D compared to those without hypovitaminosis D (7). Moreover, patients with AD have lower serum 25OHD concentrations compared to those without AD (19). Such association was found both in the severe (20) but also in the prodromal stages of AD (21), just as if hypovitaminosis D contributed to the process of neurodegenerative dementia. Consistently, cohort studies confirmed that hypovitaminosis D precedes and predicts the onset of AD (7, 9). As a consequence, an international expert consensus concluded that hypovitaminosis D increased the risk of cognitive decline and dementia in older patients (8).

Mechanisms linking hypovitaminosis D to delirium are not fully elucidated. Vitamin D receptors were found in neurons and glial cells from brain areas that are essential to cognitive function (temporal cingular and orbital cortex, thalamus, nucleus accumbens, stria terminalis and amygdala) (22). By modifying the gene expression of various proteins, vitamin D modulates some effects that could influence neurophysiology and neuroprotection (4). With regards to neurophysiology, vitamin D regulates neurotrophic agents and controls cell differentiation and maturation (23). Vitamin D also regulates the gene expression of various neurotransmitters including acetylcholine, dopamine and serotonin (6). With regards to neuroprotection, vitamin D limits inflammatory changes associated with aging in hippocampus (24), prevents the accumulation of Aβ peptides by stimulating phagocytosis (25) and blood-brain barrier efflux transport (26). Moreover, vitamin D is involved in calcium homeostasis by regulating calcium channels (27). Vitamin D also protects the brain against glutamate toxicity (28) through anti-oxidant and anti-ischemic effects. As a consequence, hypovitaminosis D is associated with changes in brain volume (29), vascularization (30) and metabolism (31); all changes that may explain the greater risk of delirium in the case of hypovitaminosis D. Nevertheless, causality could not be deducted from our observational study, and delirium may actually be the expression of an altered cognitive status responsible for hypovitaminosis D because of disability and subsequent decreased food intakes and sun exposure.

We found a positive association between the number of acute diseases and delirium. This association could be explained on the one hand by the multifactorial origin of the delirium; most patients with delirium having multiple triggering factors independently of any previous major neurocognitive disorder (32). On the other hand, it is also possible that acute diseases were the consequences of delirium (11).

Our study presents some limitations. First, the case-control design of our study is less robust than a prospective longitudinal cohort study. Second, the CAM is a screening rather than a diagnostic tool despite very high metrological performance (14). Because of its 99% specificity (14), we assume all included patients effectively suffered from delirium. Third, our results cannot be generalized to all older adults due to the unicentric recruitment and the relatively limited number of participants. Fourth, although we were able to control for important characteristics that could modify the associations, residual potential confounders might still be present such as the ApoE genotype. Fifth, because 90% of frail elderly patients admitted into our hospital geriatric acute care unit suffer from major neurocognitive disorders, we did not use chronic neurocognitive disorders as a potential confounder here. Last, the use of an observational design precludes from inferring any causal inference.

## Conclusions

We were able to report an association between serum 25OHD concentration and delirium in older adults hospitalized in a geriatric acute care unit, especially below 30 nmol/L. This result should be confirmed in larger longitudinal studies, preferentially on a variety of adult populations. Nevertheless, it provides new clues for better understanding the involvement of vitamin D in neurocognitive decline and acute failure illustrated by delirium. It also provides a scientific basis for conducting clinical trials to test the efficacy of vitamin D supplementation to prevent or improve the prognosis of delirium in older patients with initial serum 25OHD < 30 nmol/L.

## Data Availability Statement

The datasets generated for this study are available on request from the corresponding author after notification and authorization of the competent authorities.

## Ethics Statement

The studies involving human participants were reviewed and approved by Ethical committee of Angers University Hospital. No patient and/or relatives objected to the use of their health, functional and biological data for research purposes.

## Author Contributions

CA has full access to all data in the study, takes responsibility for the data, the analyses and interpretation, and has the right to publish any or all data, separate and apart from the attitudes of the sponsors, obtained funding, statistical expertise, administrative, technical, or material support, and study supervision. JC and CA: study concept and design. JC: acquisition of data. JC, GS, SK, DS-R, and CA: analysis and interpretation of data. JC, GS, and CA: drafting of the manuscript. SK, DL, and DS-R: critical revision of the manuscript for important intellectual content. All authors contributed to the article and approved the submitted version.

## Conflict of Interest

The authors declare that the research was conducted in the absence of any commercial or financial relationships that could be construed as a potential conflict of interest.
